# Meta-analysis of KAP toward COVID-19 in Chinese residents

**DOI:** 10.3389/fpubh.2024.1279293

**Published:** 2024-03-01

**Authors:** Jie Deng, Yu Fang, QiaoLing Wang, Yanyan Tian, Shumin Wang, Yuting Yang, Dongdong Yang, Songzhe Li

**Affiliations:** ^1^Hospital of Chengdu University of Traditional Chinese Medicine, Chengdu, Sichuan, China; ^2^Sports Medicine and Rehabilitation College of North Sichuan Medical College, Nanchong, Sichuan, China

**Keywords:** COVID-19, health knowledge, attitudes, practice, pandemics, Chinese people, meta-analysis

## Abstract

**Background:**

During the coronavirus disease-2019 (COVID-19) pandemic, there have been many studies on knowledge, attitudes, and practices (KAP) toward prevention of COVID-19 infection in China. Except for symptomatic treatment and vaccination, KAP toward COVID-19 plays an important role in the prevention of COVID-19. There is no systematic evaluation and meta-analysis of KAP toward COVID-19 in China. This study is the earliest meta-analysis of KAP toward COVID-19 in China’s general population. Hence, this systematic review aimed to summarize the knowledge, attitudes, and practices (KAP) of Chinese residents toward COVID-19 during the pandemic.

**Methodology:**

Following the PRISMA guidelines, articles relevant to COVID-19 KAP that were conducted among the Chinese population were found in databases such as Scopus, ProQuest, PubMed, EMbase, Web of Science, Cochrane Library, China Biology Medicine, China National Knowledge Infrastructure, CQVIP, Wanfang and Google Scholar. A random-effect meta-analysis is used to summarize studies on knowledge, attitudes, and practice levels toward COVID-19 infection in China’s general population.

**Results:**

Fifty-seven articles published between August 2020 and November 2022 were included in this review. Overall, 75% (95% CI: 72–79%) of Chinese residents had good knowledge about COVID-19, 80% (95% CI: 73–87%) of Chinese residents had a positive attitude toward COVID-19 pandemic control and prevention (they believe that Chinese people will win the battle against the epidemic), and the aggregated proportion of residents with a correct practice toward COVID-19 was 84% (95% CI: 82–87%, *I*^2^ = 99.7%).In the gender subgroup analysis, there is no significant difference between Chinese men and Chinese women in terms of their understanding of COVID-19. However, Chinese women tend to have slightly higher levels of knowledge and a more positive attitude toward the virus compared to Chinese men. When considering the urban and rural subgroup analysis, it was found that Chinese urban residents have a better understanding of COVID-19 compared to Chinese rural residents. Interestingly, the rural population displayed higher rates of correct behavior and positive attitudes toward COVID-19 compared to the urban population. Furthermore, in the subgroup analysis based on different regions in China, the eastern, central, and southwestern regions exhibited higher levels of knowledge awareness compared to other regions. It is worth noting that all regions in China demonstrated good rates of correct behavior and positive attitudes toward COVID-19.

**Conclusion:**

This study reviews the level of KAP toward COVID-19 during the pandemic period in China. The results show that the KAP toward COVID-19 in Chinese residents was above a favorable level, but the lack of translation of knowledge into practice should be further reflected on and improved. A subgroup analysis suggests that certain groups need more attention, such as males and people living in rural areas. Policy makers should pay attention to the results of this study and use them as a reference for the development of prevention and control strategies for major public health events that may occur in the future.

**Systematic Review Registration:**

https://www.crd.york.ac.uk/PROSPERO/display_record.php?RecordID=348246, CRD42022348246.

## Introduction

COVID-19 began in 2019 and spread around the world at a rapid speed. From March 11, 2020, when the World Health Organization declared a global COVID-19 pandemic (1), our world had been changed dramatically by COVID-19 (2, 3). The World Health Organization reported that as of April 28, 2023, there had been a total of 686,902,858 confirmed cases and 6,862,681 deaths worldwide (4). As of April 28, 2023, there had been 216,456,444 confirmed cases and 1,544,221 deaths in China (5). As of January 8, 2023, China had adopted a “dynamic zero-case” policy for epidemic prevention, which includes and is not limited to: restricting the movement of people, confining them to their houses, working remotely, closing public places, closing schools, and prohibiting gatherings (6). The mortality rate of COVID-19 in China declined from ~2.81% in February 2020 to ~0.12% in October 2022, which is 1.2 times that of influenza (7). On January 8, 2023, China fully relaxed the control of COVID-19, with COVID-19 infection classified as a “Class B infectious disease” instead of “Class A infectious disease” (8). The new prevention and control policy has led to significant changes in the lives of the Chinese people, affecting both their physical health and their mental health.

No doubt, COVID-19 pandemic is a highly contagious, pathogenic viral infection that has spread globally at an unprecedented rate. The most important preventive measures against this disease include the use of antiseptics, the use of face masks, social distancing, and vaccinations (9–12). Each country adopts different prevention policies, resulting in different morbidity and mortality rates among its citizens (13, 14). The mortality rate of the disease varies between countries, with reported mortality rates ranging from 2 to 5% (15, 16). Different perceptions of a disease affect people’s attitudes and practices (17). Negative and inappropriate attitudes and practices increase the risk of disease and death, as well as psychological disorders such as worry, concern, and fear of the disease (18, 19). Given the significant impact of COVID-19, numerous related studies have been conducted around the world. Among them, the KAP, a survey-based study program, can provide insights into people’s knowledge, attitudes, and practices (20, 21), which allows for a more comprehensive understanding of the general population’s perceptions of this disease and potential risk factors, and may thus help to achieve the results of planned behaviors (22, 23).

As China eases its COVID-19 prevention and control measures, it is crucial to emphasize the importance of preventing COVID-19 through comprehensive knowledge, a positive attitude, and appropriate behavior. Given the importance of this issue, it is necessary to perform a retrospective analysis of studies on KAP and summarize the results, which will provide solid evidence for better management of the disease by policy makers in China (24, 25). Therefore, this study aims to conduct a systematic review to synthesize the available evidence on KAP toward COVID-19 in China’s general population.

## Methods

### Registration and protocol

This systematic evaluation utilizes the Protocol of Preferred Reporting Items for Systematic Reviews and Meta-Analyses (PRISMA) as a guidance, including Sample, Phenomenon of Interest, Design, Evaluation, and Research Type (SPIDER), and the Population, Intervention, Comparison, and Outcome (PICO) tool to construct the research question. The systematic evaluation program is registered with PROSPERO (CRD42022348246).

### Information sources, search strategy, and study selection

We searched English and Chinese papers finalized between August 1, 2020 and November 30, 2022, and published between August 2020 and November 2022. Two researchers systematically searched Scopus, ProQuest, PubMed, EMbase, Web of Science, Cochrane Library, China Biology Medicine (CBM^1^), China National Knowledge Infrastructure (CNKI^2^), CQVIP^3^ and Wanfang.^4^ We also searched for other studies including gray literatures through Baidu Scholar and Google Scholar. The main keywords of the search strategy were “COVID-19,” “SARS-CoV-2,” “infection, SARS-CoV-2,” “2019 novel coronavirus disease,” “2019 novel coronavirus infection,” “nCoV,” “2019-Novel nCoV,” “2019-nCoV,” “nCoV 2019,” “infections,” “COVID-19 virus,” “Novel Coronavirus*,” “Severe Acute Respiratory Syndrome Coronavirus Type 2 Infection,” “Coronavirus Disease 2019,” “COVID-19 pandemic,” “SARS-COV-2,” “SARS-COV2,” “sars-coronavirus-2,” “knowledge,” “perception,” “awareness,” “consciousness,” “attitude,” “action” and “KAP.” The search strategies for PubMed and CNKI databases are shown in Supplementary Table S1. The search results are stored and managed through Endnote software.

### Overview of articles – systematic literature search through databases

We searched the following Chinese databases: CNKI, CQVIP, CBM. Details of literature search is as follows: CNKI: 3204 articles; CQVIP: 17 articles; CBM: 854 articles, with a total of 4,075 articles; 3,564 duplicate articles were filtered out through the Endnote literature management software (CNKI: 2,806 articles; CQVIP: 16 articles; CBM: 742 articles) and 511 Chinese database articles were identified. Finally, 141 articles were selected after excluding the articles with non-Chinese respondents and with other study methods than cross-sectional method through the title and abstract reading. We searched the following foreign language databases: Web of science, Pubmed, Embase, Sopus, Proquest, Cochrame and other databases, with a total of 15,266 articles. Details of literature search is as follows: Web of science: 4133 articles; Pubmed: 5030 articles; Embase: 2784 articles; Sopus: 240 articles; Proquest: 197 articles; Cochrame: 2882 articles. 4,585 articles (938 reviews, 3,647 case studies) were identified after 10,681 duplicate articles were filtered out through the Endnote literature management software. Hundred and twenty-two articles were selected after excluding articles with non-Chinese subjects by title and abstract screening.

**Figure 1:**
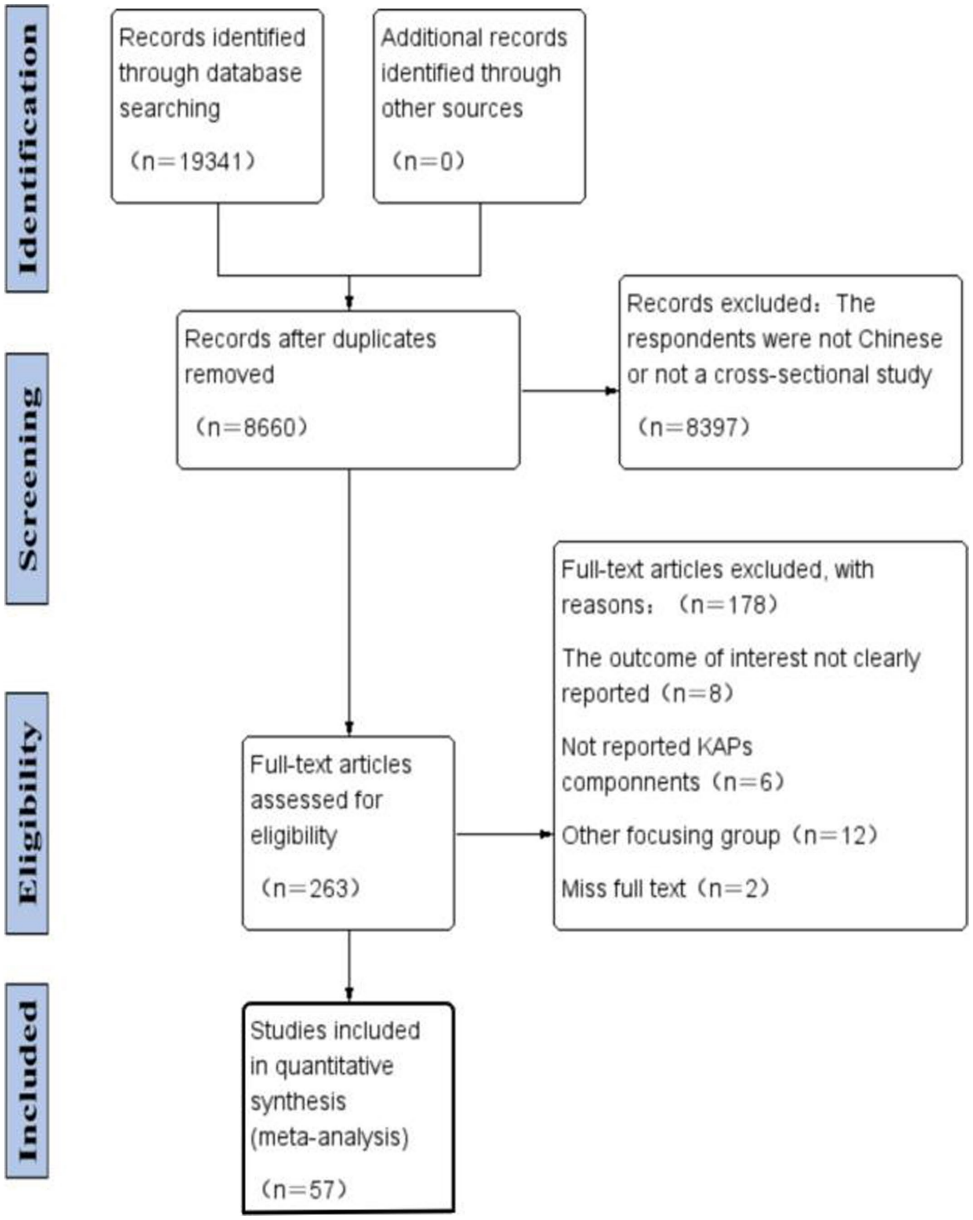
PRISMA flow diagram of literature search and study selection.

A total of 263 articles were included for full-text reading. After downloading and reading the full-texts, 178 articles whose study population were not Chinese residents were excluded, and 85 articles were selected. Finally, 57 articles were included in literature evaluation after excluding the articles with incomplete results of KAP studies (Figure 1).

### Inclusion criteria

This Study included studies reporting any form of quantitative assessment/measurement/evaluation of KAP toward COVID-19 in the general population of China. There were no restrictions on the age, sex, race or health status of subjects, or the duration of studies. Only published articles with full text (in English and Chinese) published between August 1, 2020 and November 30, 2022 were included.

### Exclusion criteria

Studies conducted only on certain groups of people, such as health care workers, medical students, pregnant women, or people with co-morbidities, were excluded. In addition, brief reports, case reports, abstracts, letters, editorials, and study document copies were excluded. Articles that met any of the following exclusion criteria were not considered as eligible full text: (1) abstracts not related to the full text, (2) articles with insufficient KAP studies, (3) reviews or meta-analyses, (4) letters to the editor, (5) studies developed on other continents, and (6) high-bias risk studies based on the Review Manager 5.4 tool.

### Study selection

To eliminate repetitive studies, articles retrieved from database (*n* = 19,341) were exported to the reference manager Zotero and Excel 2013. After carefully removing duplicate articles (*n* = 10,681), the titles and abstracts of the remaining 8,660 studies were screened. Articles with abstract data and reports that were consistent with our study topic, i.e., cross-sectional studies of knowledge, attitudes, and practices toward COVID-19 in the general population of China, were selected. Based on the inclusion criteria as well as the exclusion criteria, we performed a free-access study selection and a related study selection. Through title and abstract screening, 263 articles were selected for full-text reading. Two researchers separately performed the analysis of the full-text articles and ultimately selected the articles that met all the criteria. When the two researchers had different opinions, any disagreements should have been resolved through discussion and negotiation with a third researcher. Based on the eligible criteria, 57 articles were ultimately screened and included in this review (Figure 1).

### Quality assessment of the included studies

After excluding duplicate articles, two researchers assessed the quality of the included studies separately and critically based on the Joanna Briggs Institute checklist developed for cross-sectional studies (26). Rintala et al. (27), Ogutu et al. (28), and Pagan et al. (29) demonstrated that it is a valuable tool for testing and assessing the quality of observational studies. This checklist consists of eight straightforward questions covering topics such as sample inclusion criteria, study population and setting details, validity and reliability, measurement criteria for conditions, confounding variables, and statistical analyses (27–29). Answers to each question include Yes, No, Unclear, and Not applicable. Two researchers (Yu Fang and Qiaoling Wang) assessed the risk of bias separately. When the two authors had different opinions, the third author (Jie Deng) should have made the final decision (see Supplementary Tables S2, S3).

The overall mean score of the included studies was 5.35 according to the JBI quality assessment checklist. Of these, 33 studies (58%) were rated as good quality (score ≥ 6) and 24 studies (42%) were rated as moderate quality (score 3–5). None of the studies scored on questions 5 and 6, which was associated with a failure to identify and address confounders in the study process (for details, see Supplementary Tables S2, S3).

### Data extraction

Two researchers (Yu Fang and Jie Deng) extracted data based on the full text of the articles separately and entered them into an Excel spreadsheet template. The extracted data included author, year of publication, article title, population classification, sample source, study design, data collection method, sample size, gender percentage, standard deviation or range of age, and results related to the model components of the KAPs. (The overall mean proportion of each KAP component was calculated to obtain the knowledge, attitudes, and practices of participants in each study.) In addition, we extracted the proportion of specific content to each KAP component from the included studies. Any disagreements that arose during data extraction were resolved through discussion and negotiation. When necessary, we contacted study authors to locate missing data. Potential disagreements were resolved through negotiation with a third researcher (Qiaoling Wang).

### Data analysis

Data exported from Excel spreadsheets were analyzed using STATA version 17.0. Study heterogeneity was evaluated using the *I*^2^ statistic (%), where 25, 50, and 75% represented low, moderate, and severe heterogeneity, respectively. Due to high heterogeneity, meta-analysis was conducted using a random-effect model with the results presented on forest plots. Additional subgroup analyses were performed for rural/urban areas, gender, and geographic divisions. Publication bias was assessed using Egger’s regression test.

## Results

### Characteristics of the included studies

**Figure 2:**
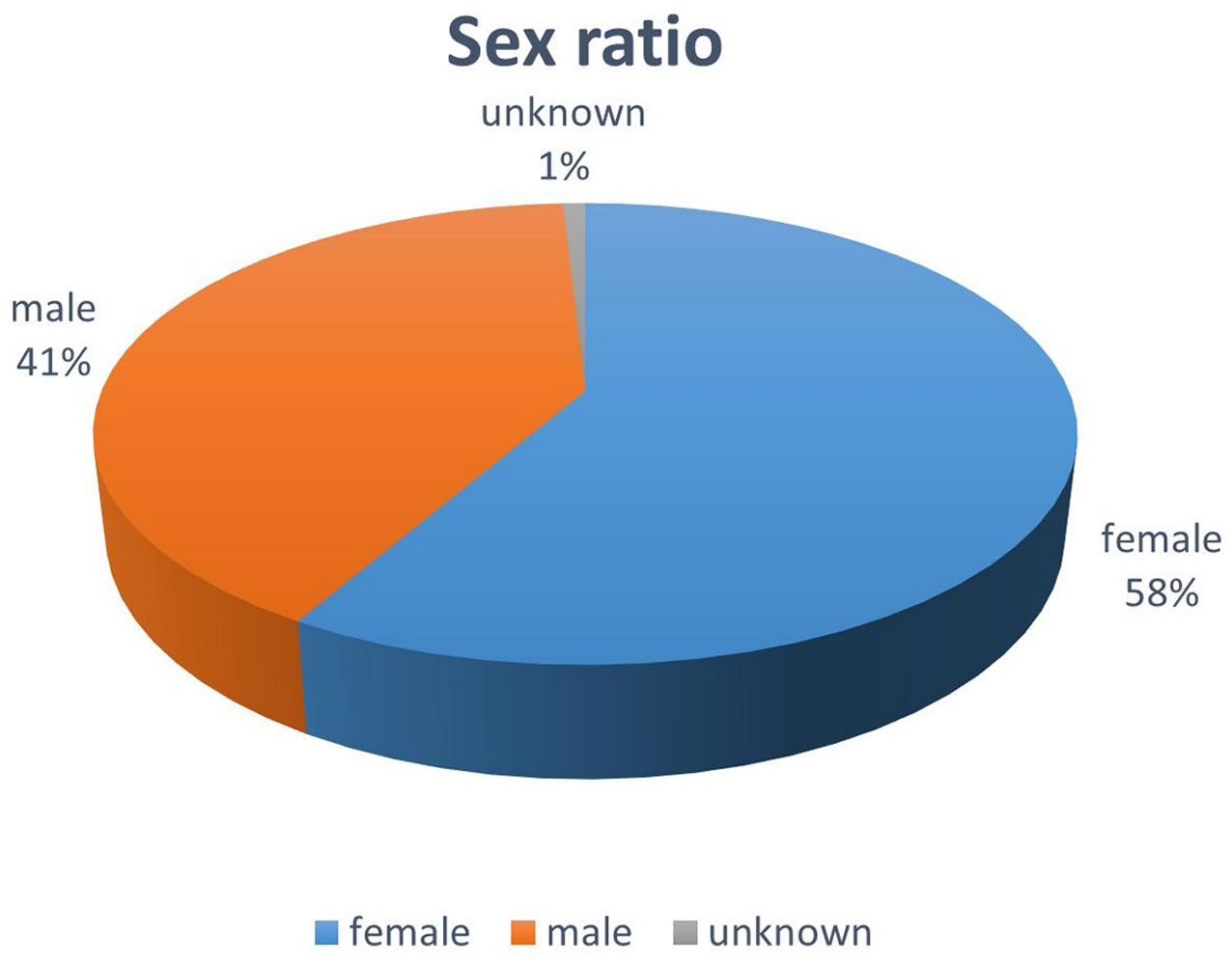
Study the sex ratio in the sample.

**Figure 3:**
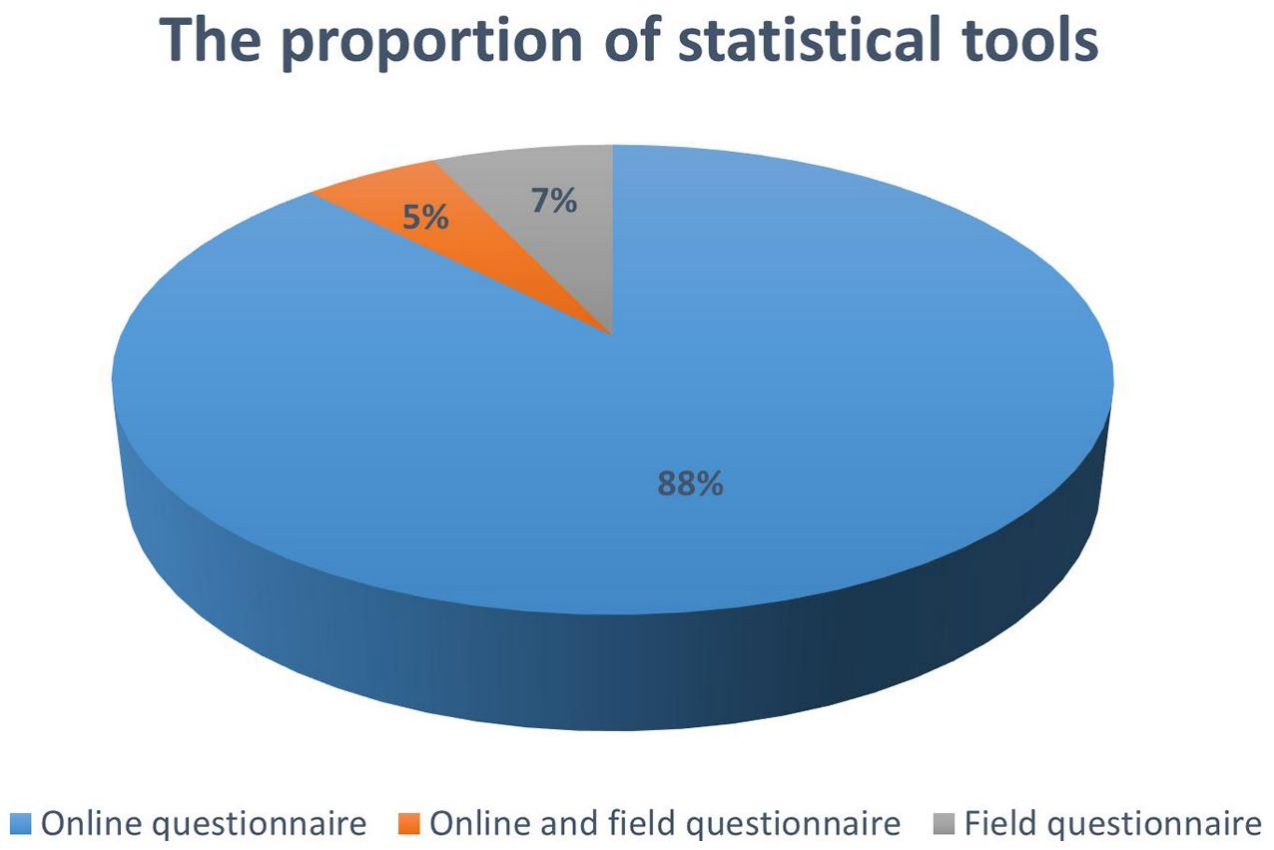
The proportion of survey methods used in the study sample.

Overall description of the included studies: There were a total of 476,518 subjects in all of the included studies (*n* = 57), including 194,552 males and 277,829 females, and 3 studies without gender statistics had 4,137 subjects (Figure 2). Study sample sizes ranged from 130 to 162,523. All respondents were Chinese citizens, and all studies were conducted in China, with sample sources from various regions of China. There were 33 articles in 2020, 19 articles in 2021 and 5 articles in 2022. The main data collection methods used include online questionnaires (*n* = 50), offline questionnaires (*n* = 4), and combined online and offline questionnaires (*n* = 3) (Figure 3).

### Measurement used in the included studies

This study was conducted on the basis of the score rate, mean standard deviation of Chinese residents’ knowledge, attitudes and practices toward COVID-19. All of the included studies contained either the score rate results of knowledge, attitudes, and practices toward COVID-19 or the mean and standard deviation results of knowledge, attitudes, and practices toward COVID-19. For the articles with study results expressed as score rate, the standard used for assessment is as follows: poor (0–60%), moderate (60–70%), good (70–85%), and excellent (85–100%). For the articles with study results expressed as mean standard deviation, the overall KAP was not assessed due to the differences in total scores of knowledge, attitudes and practices toward COVID-19 in each article. Their findings were mainly explored for subgroup analysis.

### Analyze data results

#### Results of Chinese residents’ knowledge about COVID-19

**Figure 4:**
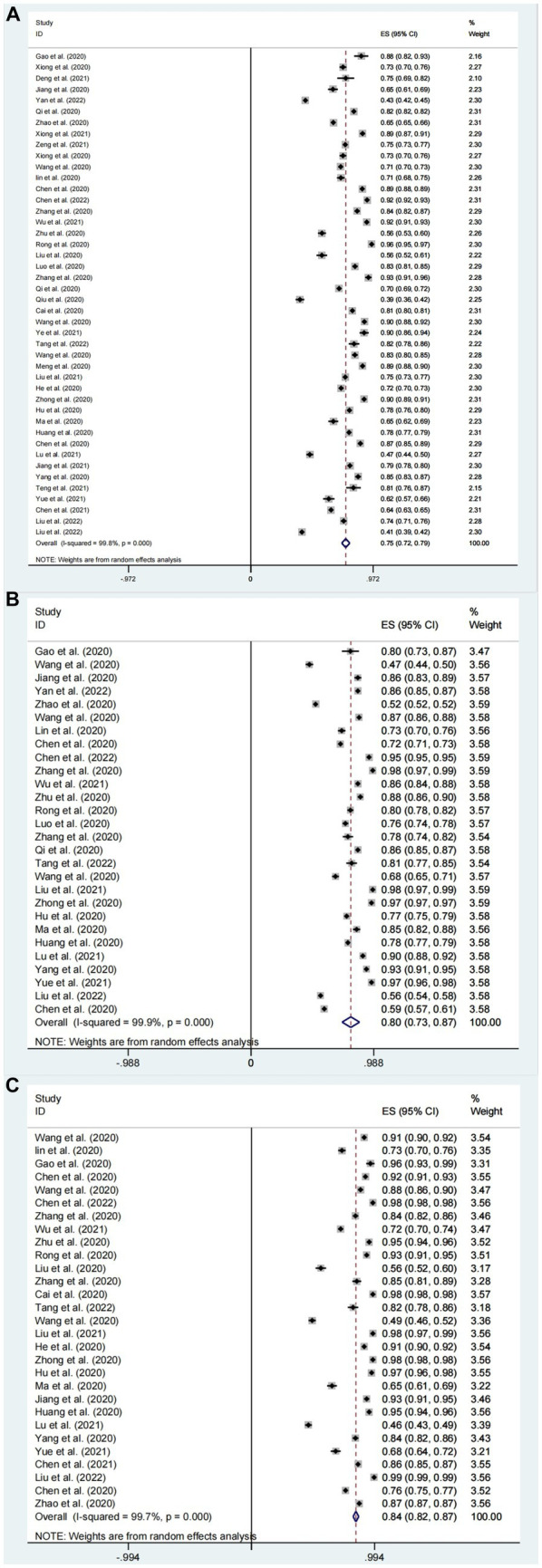
Knowledge **(A)**, attitudes **(B)**, and behavior **(C)** awareness rates of Chinese people about COVID-19 forest map.

The aggregated proportion of Chinese residents with knowledge about COVID-19 (*n* = 44) was 75% (95% confidence interval [CI]: 72–79%, *I*^2^ = 99.8%). Substantial study heterogeneity was identified (*I*^2^ = 99.8%), and a small-study effect based on the Eggers test was absent (*p* = 0.216). This suggests that Chinese residents’ knowledge about COVID-19 is at a favorable level (Figure 4).

##### Subgroup analysis for gender

**Figure 5:**
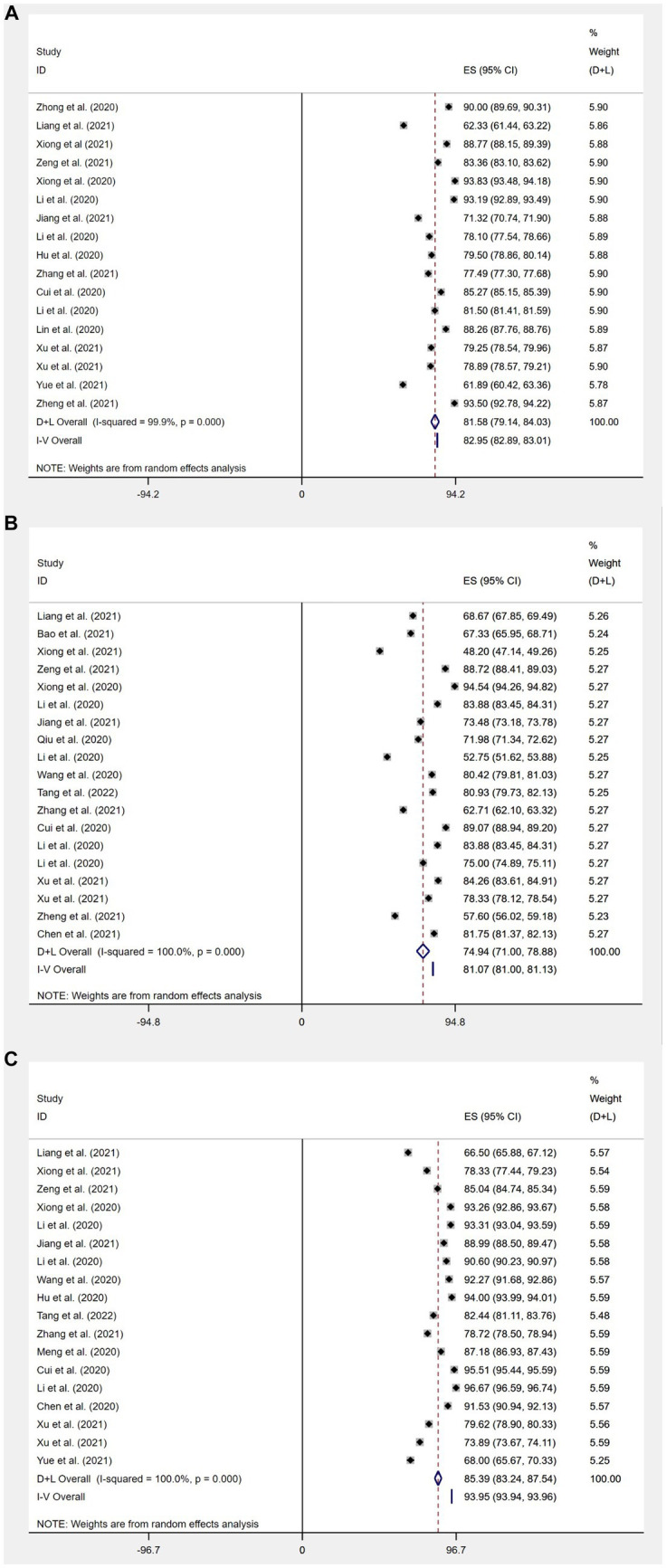
Mean standard deviation of Chinese people’s knowledge **(A)**, attitudes **(B)**, and behavior **(C)** scores on COVID-19 Forest map.

**Figure 6:**
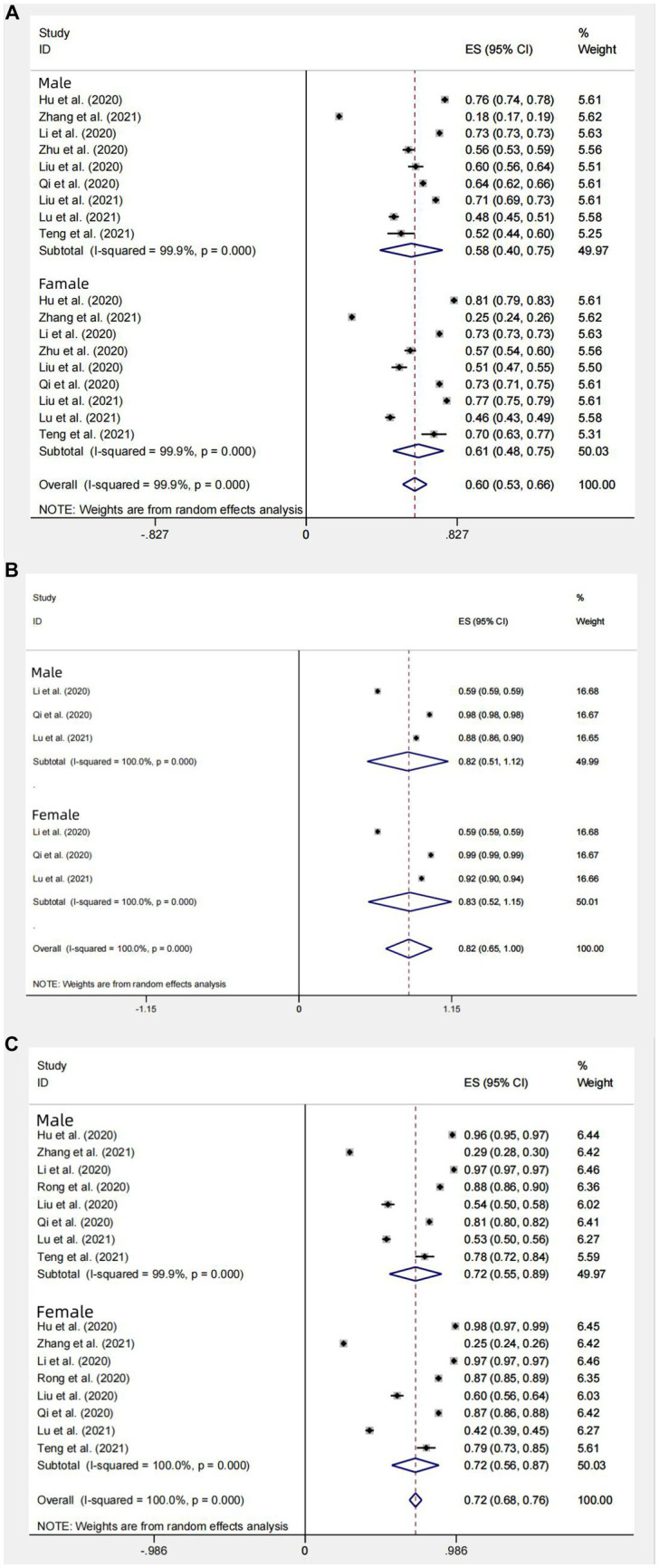
Subgroup gender analysis of knowledge **(A)**, attitudes **(B)**, and behavior **(C)** awareness of the Chinese people about COVID-19.

**Figure 7:**
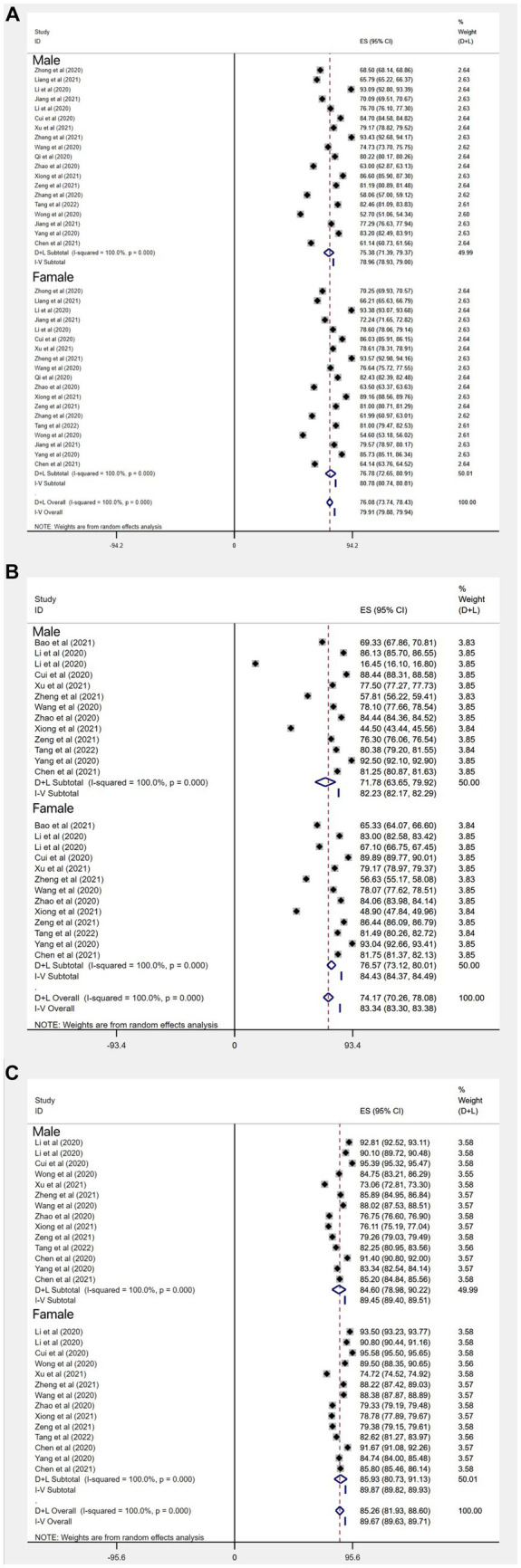
Subgroup gender analysis of the mean standard deviation of Chinese people’s knowledge **(A)**, attitudes **(B)**, and behavior **(C)** scores on COVID-19.

In the subgroup analysis for gender, COVID-19 knowledge rates for Chinese male and female residents were as follows: In the sample of studies (*n* = 9) with outcome factor expressed as rate, the knowledge rate for males was 58% (95% CI: 40–75%, *I*^2^ = 99.9%); the knowledge rate for females was 61% (95% CI: 48–75%, *I*^2^ = 100%); the overall rate for 9 studies was 60% (95% CI: 53–66%, *I*^2^ = 99.9%). In the sample of studies (*n* = 17) with outcome factor expressed as mean standard deviation (For articles that involve mean and standard deviation results as outcome factors, we conducted a thorough review of the relevant information. To ensure consistency, we selected Professor Hogg’s Introduction to Mathematical Statistics and applied a transformation formula to reanalyze the data with different baselines. For details, see Supplementary Table S4), the mean score of knowledge in 17 studies was: 81.58 (95% CI:79.14–84.03, *I*^2^ = 99.99%, *p* < 0.001) (Figure 5A). The mean score of Chinese males’ knowledge about COVID-19 was 75.38 (95% CI: 71.39–79.37, *I*^2^ = 100%, *p* < 0.001); the mean score of Chinese females’ knowledge about COVID-19 was 76.78 (95% CI, 72.65–80.91, *I*^2^ = 100%, *p* < 0.001). The results suggest that there is no significant difference between Chinese males’ knowledge and females’ knowledge about COVID-19, but the knowledge rate of Chinese females about COVID-19 is slightly better than that of Chinese males (Figures 6A, 7A).

##### Subgroup analysis for rural/urban areas

**Figure 8:**
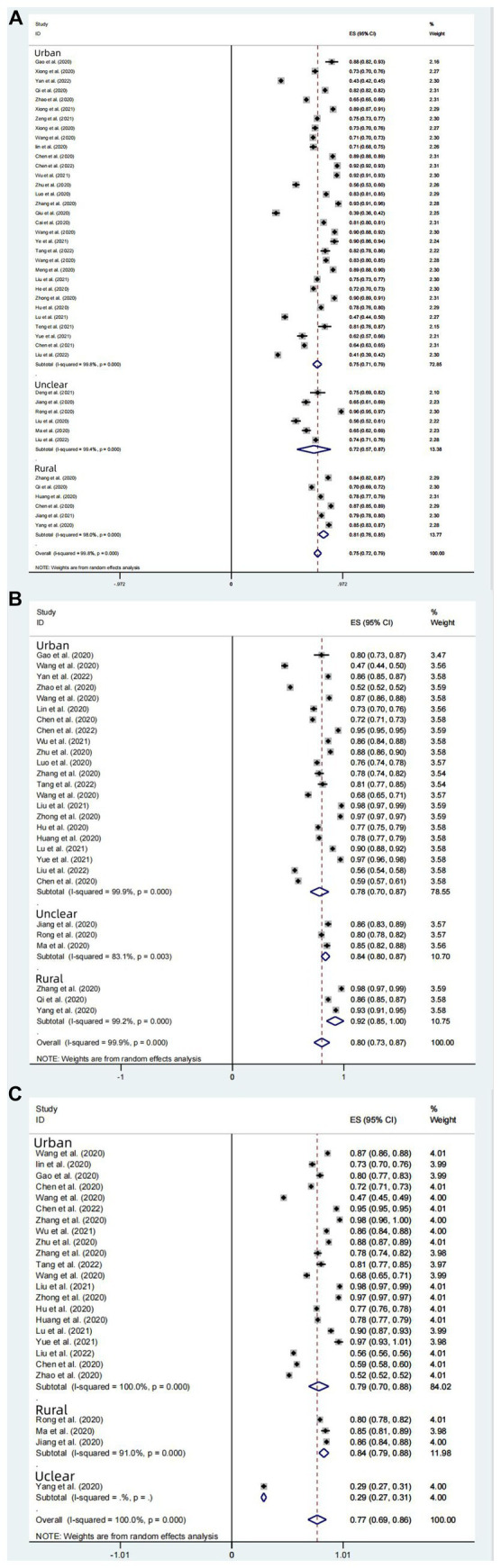
Rural–urban subgroup analysis of knowledge **(A)**, attitudes **(B)**, and behavior **(C)** awareness rates of Chinese people on COVID-19.

The COVID-19 knowledge rate of Chinese urban residents (*n* = 21) was 75% (95% CI: 71–79%, *I*^2^ = 99.8%, *p* < 0.001). The COVID-19 knowledge rate of Chinese rural residents (*n* = 6) was 72% (95% CI: 71–79%, *I*^2^ = 99.8%, *p* < 0.001). Another 6 articles (*n* = 6) did not specify urban or rural areas and showed a knowledge rate of 81% (95% CI: 76–85%, *I*^2^ = 99.8%, *p* < 0.001). Overall, the knowledge rate was higher in urban areas than in rural areas. This may be related to the fact that the dissemination of knowledge about COVID-19 is more comprehensive and thorough in urban areas than in rural areas of China (Figure 8).

##### Subgroup analysis for different regions of China

**Figure 9:**
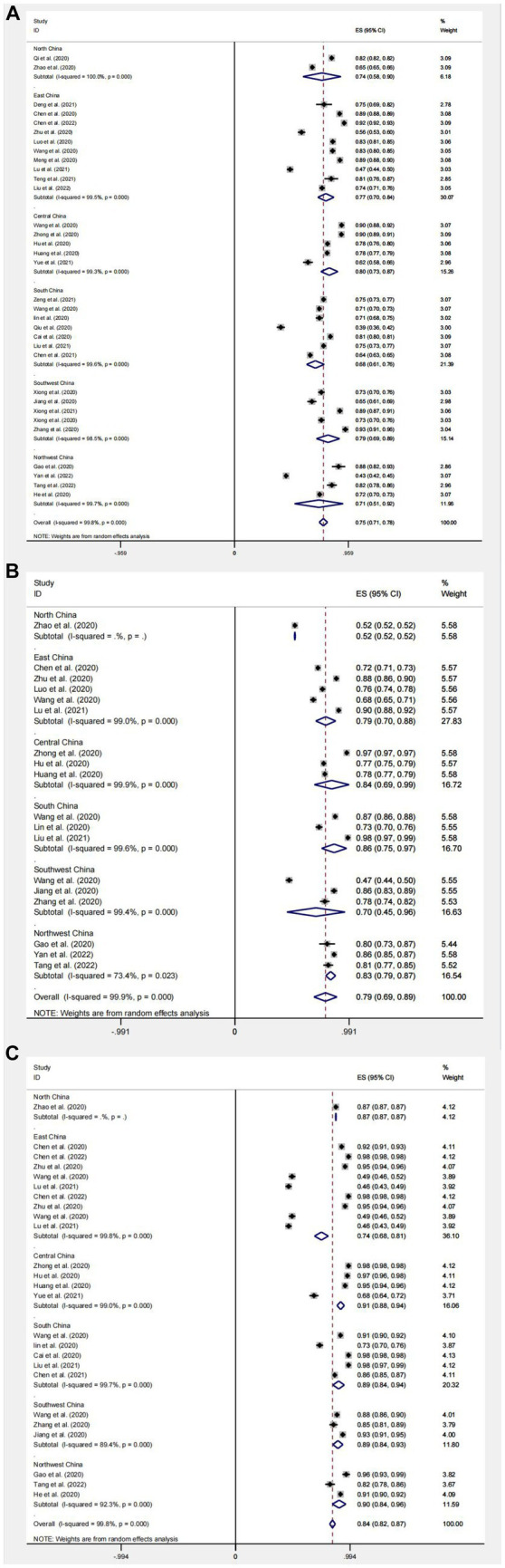
Regional subgroup analysis of knowledge **(A)**, attitudes **(B)**, and behavior **(C)** awareness rates of Chinese people on COVID-19.

Among the 33 studies included in the analysis of subgroups of different regions of China, the overall knowledge rate of the 33 studies was 75% (95% CI: 71–78%, *I*^2^ = 99.8%, *p* < 0.001); among them, the knowledge rate in North China (*n* = 2) was 74% (95% CI: 58–89%, *I*^2^ = 100%, *p* < 0.001); the knowledge rate in East China (*n* = 10) was 77% (95% CI: 70–80%, *I*^2^ = 99.5%, *p* < 0.001); the knowledge rate in Central China (*n* = 5) was 80% (95% CI: 73–87%, *I*^2^ = 99.3%, *p* < 0.001); the knowledge rate in South China (*n* = 7) was 68% (95% CI: 61–76%, *I*^2^ = 99.6%, *p* < 0.001); the knowledge rate in Southwest China (*n* = 5) was 79% (95% CI: 69–89%, *I*^2^ = 98.5%, *p* < 0.001); the knowledge rate in Northwest China was 71% (95% CI, 51–92%, *I*^2^ = 99.7%, *p* < 0.001). Overall, the knowledge rate in East, Central and Southwest China is better than other regions (Figure 9).

#### Outcomes of Chinese residents’ attitudes toward COVID-19

The aggregated proportion of Chinese residents with positive attitudes toward COVID-19 (*n* = 28) was 80% (95% CI: 73–87%, *I*^2^ = 99.9%). Substantial study heterogeneity was identified (*I*^2^ = 99.9%), and a small-study effect based on the Eggers test was absent (*p* = 0.646). This suggests that the Chinese residents’ attitudes toward COVID-19 are at a favorable level. The difference between mean standard deviations of attitudes (*n* = 23) was: 21.49 (95% CI: 18.66–24.31, *I*^2^ = 100%) (Figures 4B, 5B).

##### Subgroup analysis for gender

In three studies (*n* = 3) with attitude outcome factor expressed as rate, rate of positive attitudes toward COVID-19 in Chinese male residents was 82% (95% CI: 51–112%, *I*^2^ = 99.9%, *p* < 0.001); rate of positive attitudes toward COVID-19 in Chinese female residents was 83% (95% CI: 52%–115, *I*^2^ = 100%, *p* < 0.001); the overall rate in 3 studies was 82% (95% CI: 65–100%, *I*^2^ = 100%, *p* < 0.001). Among the 19 studies (*n* = 19) with attitude outcome factor expressed as mean standard deviation, the total score of attitudes in 14 studies was: 74.94 (95%CI: 71.00–78.88, *I*^2^ = 100%, *p* < 0.001) (Figure 5B). The score of the Chinese male residents’ attitudes toward COVID-19 was: 71.78 (95% CI: 63.65–79.92, *I*^2^ = 100%, *p* < 0.001); the score of Chinese female residents’ attitudes toward COVID-19 was 76.57 (95% CI, 73.12–80.01, *I*^2^ = 100%, *p* < 0.001). The results suggest that both the rate and score of positive attitudes toward COVID-19 in Chinese females is slightly better than that in Chinese males (Figures 6B, 7B).

##### Subgroup analysis for rural/urban areas

The rate of positive toward COVID-19 was 78% (95% CI: 70–87%, *I*^2^ = 99.9%, *p* < 0.001) in Chinese urban resident group (*n* = 22); the rate of positive attitudes toward COVID-19 was 84% (95% CI: 80–87%, *I*^2^ = 83.1%, *p* < 0.001) in Chinese rural resident groups (n = 3). Another 3 articles (*n* = 3) did not specify urban or rural areas and showed that the rate was 92% (95% CI: 85–100%, *p* < 0.001). Overall, the rate of positive attitudes in rural areas is higher than that in urban areas, which may be due to the significantly higher sample size in the urban group compared to the rural group (Figure 8B).

##### Subgroup analysis for different regions

Among the 18 studies included in the analysis of subgroups of different regions of China, the overall rate of positive attitudes toward COVID-19 in the 18 studies was 79% (95% CI: 69–89%, *I*^2^ = 99.9%, *p* < 0.001), The rate of positive attitudes toward COVID-19 in North China (*n* = 1) was 52% (95% CI: 52–52%, *p* < 0.001). The rate of positive attitudes toward COVID-19 in East China (*n* = 5) was 79% (95% CI: 70–88%, *I*^2^ = 99.0%, *p* < 0.001); the rate of positive attitudes toward COVID-19 in Central China (*n* = 3) was 84% (95% CI: 69–99%, *I*^2^ = 99.9%, *p* < 0.001); the rate of positive attitudes toward COVID-19 in South China (*n* = 3) was 86% (95% CI: 75–97%, *I*^2^ = 99.6%, *p* < 0.001); the rate of positive attitudes toward COVID-19 in Southwest China (*n* = 3) was 70% (95% CI: 45–96%, *I*^2^ = 99.4%, *p* < 0.001); the rate of positive attitudes toward COVID-19 in Northwest China (*n* = 3) was 83% (95% CI, 79–87%, *I*^2^ = 73.4%, *p* < 0.001). Overall, rates of positive attitudes toward COVID-19 in different regions of China were above a favorable level, except for North China, where the rate at positive attitudes toward COVID-19 were only 52%, probably due to a small sample size (Figure 9B).

#### Outcomes of Chinese residents’ practice level toward COVID-19

The aggregated proportion of Chinese residents’ practices for correctly responding to COVID-19 was 84% (95% CI: 82–87%, *I*^2^ = 99.7%). Eggers’ test (*p* < 0.001) results suggest publication bias. The mean standard deviation between scores of Chinese residents’ practices for correctly responding to COVID-19 was (*n* = 23): 26.23 (95% CI: 24.67–27.8, *I*^2^ = 100%, *p* < 0.001). This suggests that Chinese residents’ practices for COVID-19 are at a favorable level (Figures 4C, 5C).

##### Subgroup analysis for gender

In the 8 studies (*n* = 8) with gender subgroups and outcome expressed as rate, their overall aggregated proportion of practices for correctly responding to COVID-19 was 72% (95% CI: 68–76%, *I*^2^ = 100%, *p* < 0.001); the aggregated proportion of Chinese males’ practices for correctly responding to COVID-19 was 72% (95% CI: 55–89%, *I*^2^ = 99.9%, *p* < 0.001); that of females was 72% (95% CI: 56–87%, *I*^2^ = 100%, *p* < 0.001). In the 18 studies with gender subgroups and outcome expressed as score of practices for correctly responding to COVID-19, the overall score of practices in the 18 studies was 85.39 (95% CI: 83.24–87.54, *I*^2^ = 100%, *p* < 0.001) (Figure 5C); the score of Chinese males’ practices for correctly responding to COVID-19 was 84.60 (95% CI: 78.98–90.22, *I*^2^ = 100%, *p* < 0.001); that of females was 85.93 (95% CI: 80.73–91.13, *I*^2^ = 100%, *p* < 0.001). The results suggest that there is no significant difference in Chinese males’ and females’ practices for correctly responding to COVID-19 (Figures 6C, 7C).

##### Subgroup analysis for rural/urban areas

Among the 27 studies (*n* = 27) with Chinese rural or urban residents as subjects, the rate of practices for correctly responding to COVID-19 was 79% (95% CI: 70–88%, *I*^2^ = 100%, *p* < 0.001) in urban group (*n* = 21), and 84% (95% CI: 79–88%, *I*^2^ = 91%, *p* < 0.001) in rural group (*n* = 6). Another 1 article (*n* = 1) did not specify urban or rural areas and showed that the rate was 29% (95% CI, 27–31%, *p* < 0.001). Overall, both the residents in urban and rural areas of China show favorable rates of practices for correctly responding to COVID-19. The results of this subgroup study reflected that rural groups had a higher rate of practices for correctly responding to COVID-19 practice than urban groups, which may be due to the smaller sample size in the rural group (Figure 8C).

##### Subgroup analysis for different regions

Among the 25 studies (*n* = 25) included in the subgroup analysis for different regions of China, the aggregated proportion of practices for correctly responding to COVID-19 was 84% (95% CI: 82–87%, *I*^2^ = 99.8%, *p* < 0.001);The aggregated proportion of practices for correctly responding to COVID-19 in North China (*n* = 1) was 87% (95% CI: 87–87%, *p* < 0.001); the aggregated proportion of practices for correctly responding to COVID-19 in East China (*n* = 9) was 74% (95% CI: 68–81%, *I*^2^ = 99.8%, *p* < 0.001); the aggregated proportion of practices for correctly responding to COVID-19 in Central China (*n* = 4) was 91% (95% CI: 88–94%, *I*^2^ = 99.0%, *p* < 0.001); the aggregated proportion of practices for correctly responding to COVID-19 in South China (*n* = 5) was 89% (95% CI: 84–94%, *I*^2^ = 99.7%, *p* < 0.001); the aggregated proportion of practices for correctly responding to COVID-19 in Southwest China (*n* = 3) was 89% (95% CI: 84–93%, *I*^2^ = 89.4%, *p* < 0.001); the aggregated proportion of practices for correctly responding to COVID-19 in Northwest China (*n* = 3) was 90% (95% CI: 84–96%, *I*^2^ = 92.3%, *p* < 0.001); the overall rate of correct practices was 84% (95% CI: 82–87%, *I*^2^ = 99.8%, *p* < 0.001), Overall, the rate of practices for correctly responding to COVID-19 in different regions of China is at a favorable level, with no significant difference between regions, which is attributed to the vigorous promotion and effective implementation of COVID-19 prevention and control measures in China (Figure 9C).

### Publication bias analysis

**Figure 10:**
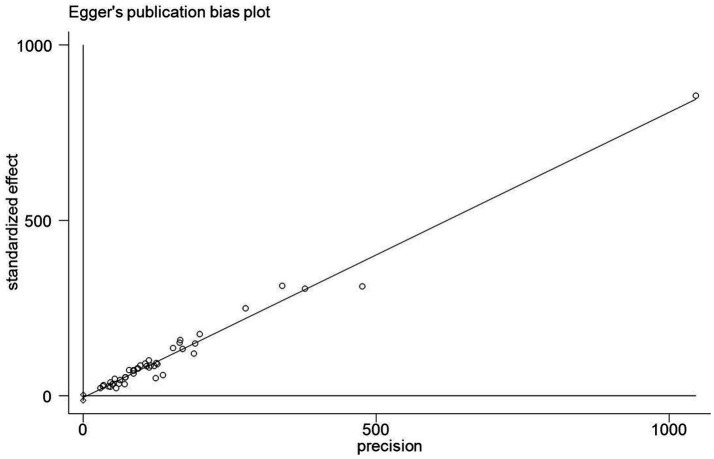
Publication bias chart of COVID-19 knowledge awareness rate in China.

**Figure 11:**
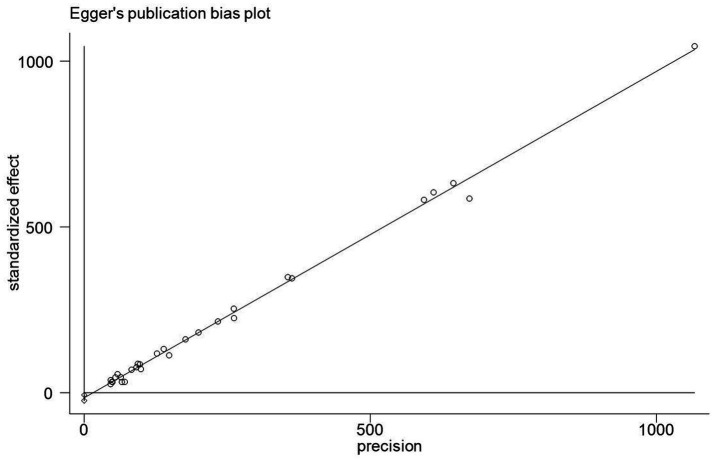
A biased analysis of the awareness rate of Chinese people on COVID-19.

**Figure 12:**
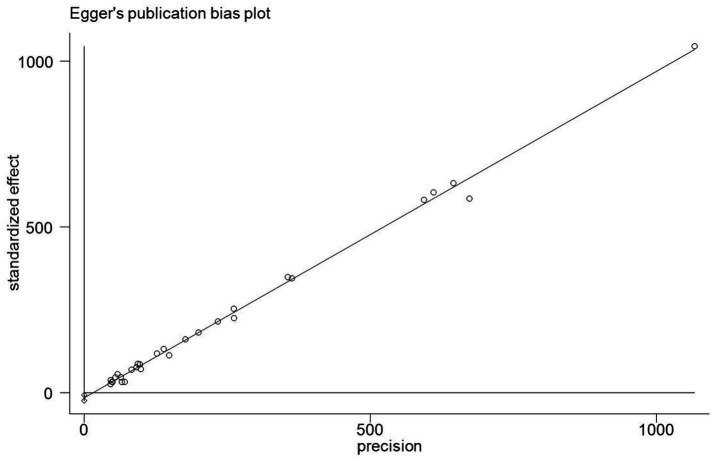
Publication bias chart of COVID-19 behavior awareness rate in China.

In this study, Egger’s linear regression test was used to assess the publication bias of KAP knowledge rate, and the results suggested that there was no significant publication bias (Figures 10–12).

### Sensitivity analysis

**Figure 13:**
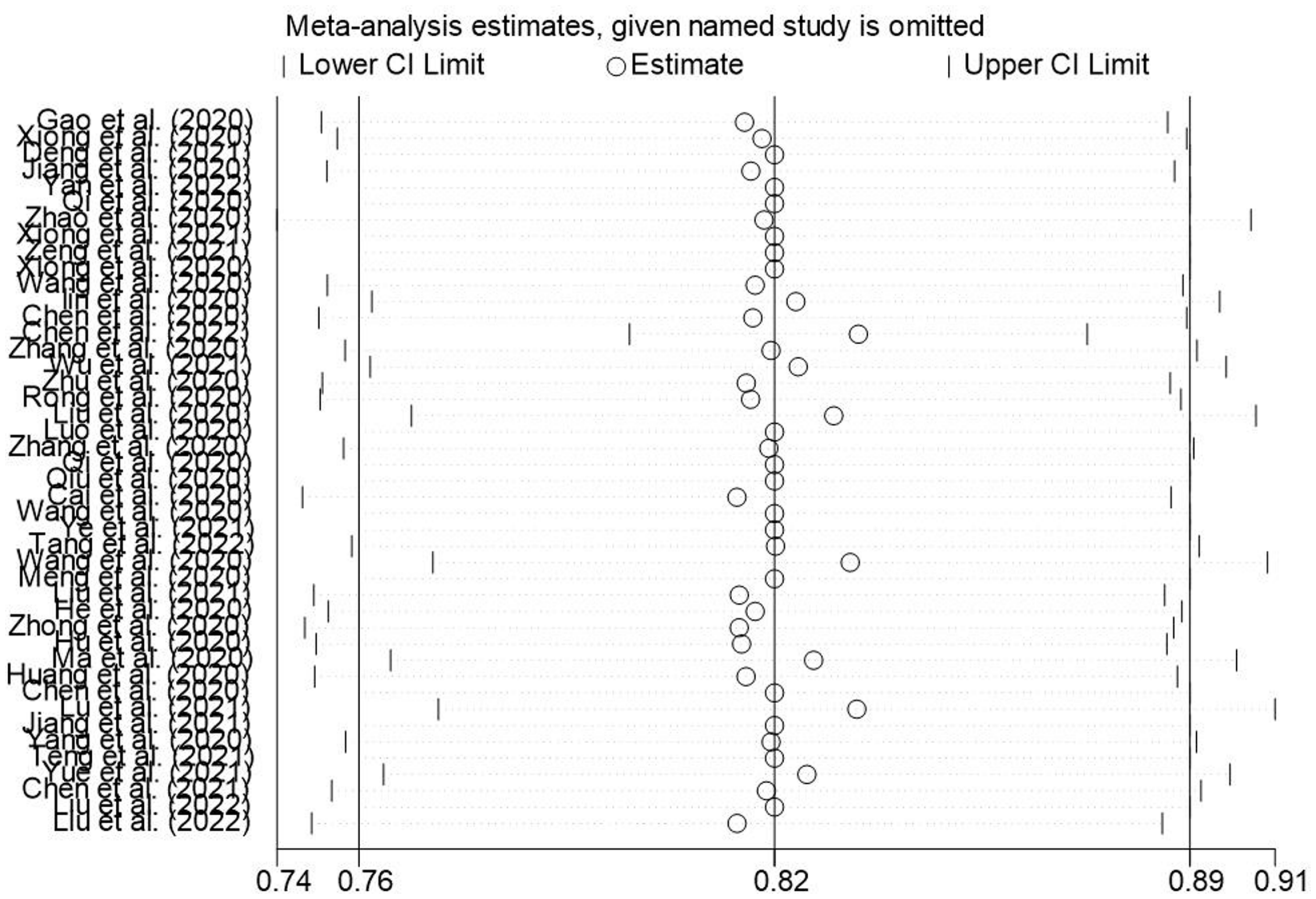
Sensitivity analysis of knowledge awareness rate of Chinese people on COVID-19.

**Figure 14:**
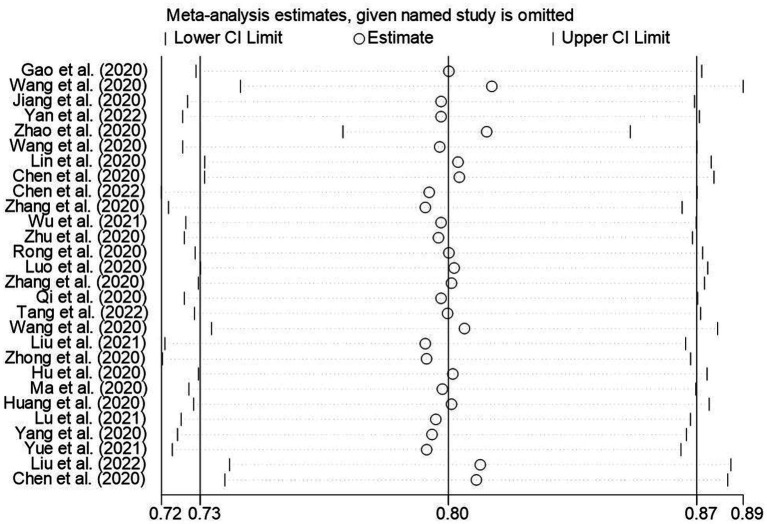
Sensitivity analysis of the awareness rate of Chinese people on COVID-19.

**Figure 15:**
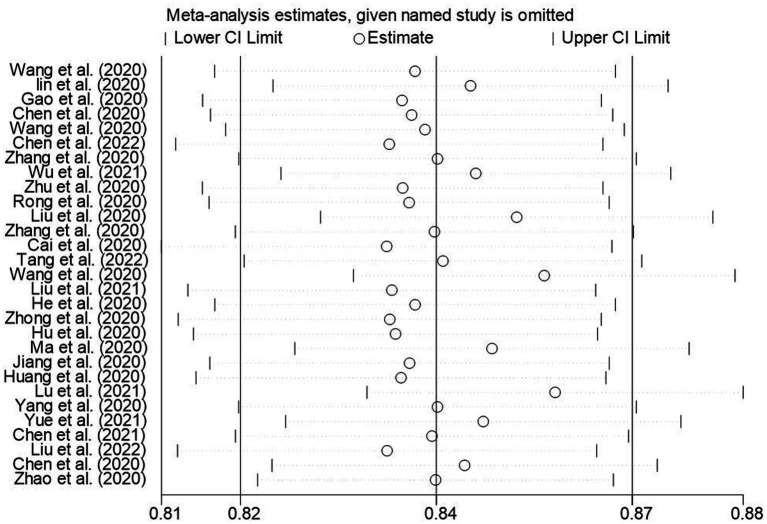
Sensitivity analysis of behavioral awareness rate of Chinese people for COVID-19.

The results of the sensitivity analysis of the knowledge, attitudes, and practices rates in this study suggested that the knowledge, attitude, and practices rates in this study were relatively stable (Figures 13–15).

## Discussion

Despite the fact that CDVID-19 pandemic has been prevailing globally for 3 years, and almost all countries are now adopting open prevention and control policies, COVID-19 is the most serious epidemic disease in this century (30). Given the fact that there are various mutations and variant strains of the COVID-19 virus (31), we still need to take effective prevention and control measures to reduce the number of severe and fatal cases. The aim of this study is to assess the knowledge, attitudes and practices to wardCOVID-19 among Chinese residents, which may assist the prevention and control authorities to adjust the epidemic prevention and control measures and tools. In the systematic evaluation and meta-analysis part of this study, relevant studies were searched and screened, and 57 relevant studies were included for meta-analysis. The overall estimates for correct answers to knowledge, good attitudes and good practices toward COVID-19 among Chinese residents in this study were 75, 80, and 84%, respectively. However, the slight difference between knowledge and practices may be due to the fact that although measures such as epidemic prevention and control education were well implemented in China, there is still a lack of knowledge dissemination about COVID-19. And the significant imbalance in the education level of China’s general population is also a contributing factor. In a large systematic evaluation and meta-analysis of KAP toward the novel COVID-19 in a worldwide general population, data was collected from 215,731 participants from 84 studies in 45 countries, and the overall correct answer estimates for knowledge, good attitudes, and good practices were 75, 74, and 70%, respectively (20). Also, a study in Bangladesh showed: The public’s perception of controlling COVID-19 is mixed, with only 44.16% (95% CI: 35.74–52.93) and 60.28% (95% CI, 49.22–70.38) believing the country would win the struggle against the pandemic and the infection will be successfully controlled, respectively (32). The KAP data for citizens of China were superior to these two studies, which may be due to the effective and consistent prevention and control efforts implemented across the whole country (33). A 2018 study on seasonal flu in East China reported that 21 and 20% of Chinese citizens were aware of seasonal flu virus or vaccination, respectively, but less than 1% of citizens reported having received a flu vaccine (34). KAP (knowledge, attitudes, and practices) toward flu infection in Chinese citizens were low (35–37), and need to be improved. The importance of vaccination was widely publicized during the COVID-19 pandemic (38, 39). During the COVID-19 pandemic in 2021, a study on KAP toward flu and vaccination among Chinese citizens participants indicated 78.7% correct answers. 73.04% of participants knew that vaccination was the most effective measure against flu infection. The percentage of participants who were willing to be vaccinated was 85.82% (40). It can be seen that the Chinese people’s knowledge about flu and vaccination had been significantly improved during the COVID-19 pandemic.

Analysis of subgroups suggested that females have higher scores for knowledge, attitudes, and practices than males. Consistent with a study of COVID-19 KAP among healthcare providers (41), similar results have been found in Southeast and South Asia studies (42). This is consistent with the findings of a meta-analysis called “the association between gender and protective behaviors in response to respiratory epidemics and pandemics” in the general population, which showed that females were more likely than males to adopt or practice preventive behaviors (e.g., washing hands, wearing face masks, and avoiding taking public transportation) (43). People living in rural areas show rate of knowledge (72%) lower than people living in cities. This is consistent with the findings of an Egyptian study (44). However, people living in rural areas show attitudes (84%) and practices (84%) higher than people living in urban areas [attitudes (78%) and practices (79%)]. A study of COVID-19 KAP among pregnant women around the world shows: pregnant women who resided in urban areas were 2.23 times more likely to have good preventive practices for COVID-19 infection compared with those who resided in rural areas, This finding contradicts the results of our study and further confirms the presence of result bias due to the limited sample size in rural areas during the analysis of rural (45). urban subgroups. The economic subregions showed insignificant differences. Central China showed the highest knowledge rate of 80% (95% CI: 73–87%). South China showed the lowest knowledge rate of 68% (95% CI: 61–76%); North China showed the lowest rate of 52% (95% CI: 52–52%); South China showed the highest rate of positive attitudes of 86% (95% CI: 75–97%); East China showed the lowest rate of practices of 74% (95% CI: 68–81%); Central China showed the highest rate of practices of 0.91 (95% CI: 88–94%), which was the highest (46). There was no significant difference between rural and urban areas, and between various economic subregions, which may be related to China’s vigorous promotion and strict COVID-19 prevention and control policies, which have resulted in high levels for COVID-19 KAP in all regions of China. Before summarizing the importance and implications of the results of this meta-analysis, some limitations should be noted. Journal articles published in English did not consider using other sources, such as preprint articles. Most included studies were conducted through online data collection methods, limiting the generalizability of the findings to the entire national population. However, as the earliest Meta-analysis evaluating the Chinese people’s KAP toward COVID-19, this study not only provides insights for the Chinese people, the Chinese government, and the health organizations, but also has the most important implications for the current long-term coexistence with COVID-19 and for diseases such as seasonal flu. First, this study helps the rest of the world to understand the level of KAP toward COVID-19 among the Chinese people, and it can help individual practitioners to design different survey programs (23). In addition, it can help to identify certain groups that need more attention, such as males, people living in rural areas, the singles, and people with lower household income. Secondly, this study suggests that the government should not only be responsible for the surveillance of epidemic diseases, but should also further promote and popularize disease prevention and control knowledge. Finally, the government should actively identify actual operating difficulties encountered during prevention and control process and solve these difficulties.

## Conclusion

This study reviews the level of KAP toward COVID-19 during the pandemic period in China. The results show that the KAP toward COVID-19 in Chinese residents was above a favorable level, but the lack of translation of knowledge into practice should be further reflected on and improved. A subgroup analysis suggests that certain groups need more attention, such as males and people living in rural areas. Policy makers should pay attention to the results of this study and use them as a reference for the development of prevention and control strategies for major public health events that may occur in the future.

## Data availability statement

The original contributions presented in the study are included in the article/Supplementary material, further inquiries can be directed to the corresponding author.

## Author contributions

JD: Data curation, Formal analysis, Methodology, Supervision, Writing – original draft, Writing – review & editing. YF: Data curation, Formal analysis, Methodology, Supervision, Writing – original draft, Writing – review & editing. QW: Data curation, Methodology, Supervision, Writing – review & editing. YT: Investigation, Writing – review & editing. SW: Investigation, Writing – review & editing. YY: Validation, Writing – review & editing. DY: Writing – review & editing, Validation. SL: Validation, Writing – review & editing.

## Footnotes

^1^http://www.sinomed.ac.cn/index.jsp

^2^https://www.cnki.net/

^3^http://lib.cqvip.com/

^4^https://new.wanfangdata.com.cn/index.html
