# Supplementary material for: Meta-analysis of KAP toward COVID-19 in Chinese residents
Source: Front Public Health. 2024 Mar 1;12:1279293. doi: 10.3389/fpubh.2024.1279293 (PMC10944655; doi:10.3389/fpubh.2024.1279293)

Supplementary Material

# Supplementary Table 1: Algorithm for study search to identify published articles on the knowledge, attitude and practice of COVID-19 among Chinese residents

|  | **Search terms** |
| --- | --- |
| **#1** | "COVID-19"[MeSH Terms] OR "SARS-CoV-2"[MeSH Terms] |
| **#2** | "COVID-19"[Title/Abstract] OR "COVID 19"[Title/Abstract] OR " Infection, SARS-CoV-2 " [Title/Abstract] OR"SARS-CoV-2 Infection" [Title/Abstract] OR "2019 Novel Coronavirus Disease"[Title/Abstract] OR" 2019 Novel Coronavirus Infection"[Title/Abstract] OR"nCoV"[Title/Abstract] OR "2019-novel nCoV"[Title/Abstract] OR "2019- ncov"[Title/Abstract] OR "2019 ncov"[Title/Abstract] OR "nCov 2019"[Title/Abstract] OR "Infection, COVID-19 Virus""[Title/Abstract] OR  "ncov19"[Title/Abstract] OR "novel coronavirus*"[Title/Abstract] OR "new coronavirus*"[Title/Abstract] OR "Severe Acute Respiratory Syndrome Coronavirus 2 Infection"[Title/Abstract] OR "coronavirus disease 2019"[Title/Abstract] OR "coronavirus disease-2019"[Title/Abstract] OR "corona-virus disease 2019"[Title/Abstract] OR "COVID-19 Pandemics"[Title/Abstract] |
| **#3** | "SARS-COV-2"[Title/Abstract] OR "SARS-COV2"[Title/Abstract] OR "SARS-Coronavirus-2"[Title/Abstract] OR "SARS-Coronavirus2"[Title/Abstract] OR "SARS-Corona-Virus-2"[Title/Abstract] |
| **#4** | "coronavirus*"[Title/Abstract] AND ("19"[Title/Abstract] OR "2019"[Title/Abstract]) |
| **#5** | "wuhan"[Title/Abstract] AND ("coronavirus"[Title/Abstract] OR "corona virus"[Title/Abstract]) |
| **#6** | #1 OR #2 OR #3 OR #4 OR #5 |
| **#7** | "Knowledge"[MeSH Terms] OR "Perception"[MeSH Terms] OR "Awareness"[MeSH Terms] OR "Consciousness"[MeSH Terms] OR "Attitude"[MeSH Terms] |
| **#8** | "knowledge"[Title/Abstract] OR "perception"[Title/Abstract] OR "awareness"[Title/Abstract] OR "consciousness"[Title/Abstract] OR "attitude"[Title/Abstract] OR "practice"[Title/Abstract] OR "action"[Title/Abstract] OR "KAP"[Title/Abstract] |
| **#9** | #7 OR #8 |
| **#10** | "China"[MeSH Terms] |
| **#11** | "China"[Title/Abstract] |
| **#12** | #10 OR #11 |
| **#13** | #6 AND #9 AND #12 |

**2.Supplement Table 2:Study quality assessment questions**

| **Risk of bias items** | | | |
| --- | --- | --- | --- |
| 1 | Were the criteria for inclusion in the sample clearly defined? | 7 | Were the outcomes measured in a valid and reliable way? |
| 2 | Were the study subjects and the setting described in detail? | 8 | Was appropriate statistical analysis used? |
| 3 | Was the exposure measured in a valid and reliable way? |  |  |
| 4 | Were objective, standard criteria used for measurement of the condition? |  |  |
| 5 | Were confounding factors identified? |  |  |
| 6 | Were strategies to deal with confounding factors stated? |  |  |

**3.Supplement Table 3:Study quality assessment scores**

| **Reference (Author, Year)** | **Q1** | **Q2** | **Q3** | **Q4** | **Q5** | **Q6** | **Q7** | **Q8** | **Total** |
| --- | --- | --- | --- | --- | --- | --- | --- | --- | --- |
| Zhong et al.(2020) | U | U | Y | Y | N | N | Y | Y | 4 |
| Gao et al.(2020) | Y | U | Y | Y | N | N | Y | Y | 5 |
| Wang et al.(2020) | Y | Y | Y | Y | N | N | Y | Y | 6 |
| Liang et al.(2021) | U | U | Y | Y | N | N | Y | Y | 4 |
| Deng et al.(2021) | Y | Y | Y | Y | N | N | Y | Y | 6 |
| Jiang et al.（2020） | U | Y | Y | Y | N | N | Y | Y | 5 |
| Yan et al.(2022) | U | Y | Y | Y | N | N | Y | Y | 5 |
| Qi et al.(2020) | U | U | Y | Y | N | N | Y | Y | 4 |
| Zhao et al.(2020) | U | U | Y | Y | N | N | Y | Y | 4 |
| Bao et al.(2021) | Y | Y | Y | Y | N | N | Y | Y | 6 |
| Xiong et al.(2021) | Y | Y | Y | Y | N | N | Y | Y | 6 |
| Zeng et al.(2021) | Y | Y | Y | Y | N | N | Y | Y | 6 |
| Xiong et al.(2020) | Y | U | Y | Y | N | N | Y | Y | 5 |
| Wang et al.(2020) | Y | Y | Y | Y | N | N | Y | Y | 6 |
| Lin et al.(2020) | Y | Y | Y | Y | N | N | Y | Y | 6 |
| Zhang et al.(2021) | Y | Y | Y | Y | N | N | Y | Y | 6 |
| Li et al.(2020) | U | U | Y | Y | N | N | Y | Y | 4 |
| Chen et al.(2020) | U | U | Y | Y | N | N | Y | Y | 4 |
| Chen et al.(2022) | Y | Y | Y | Y | N | N | Y | Y | 6 |
| Zhang et al.(2020) | Y | Y | Y | Y | N | N | Y | Y | 6 |
| Jiang et al.(2021) | Y | Y | Y | Y | N | N | Y | Y | 6 |
| Gao et al（2021） | Y | Y | Y | Y | N | N | Y | Y | 6 |
| Zhu et al.(2020) | Y | Y | Y | Y | N | N | Y | Y | 6 |
| Rong et al.(2020) | Y | U | Y | Y | N | N | Y | Y | 5 |
| Liu et al.(2020) | Y | Y | Y | Y | N | N | Y | Y | 6 |
| Luo et al.(2020) | Y | Y | Y | Y | N | N | Y | Y | 6 |
| Zhang et al.(2020) | Y | Y | Y | Y | N | N | Y | Y | 6 |
| Qi et al.(2020) | Y | U | Y | Y | N | N | Y | Y | 5 |
| Qiu et al.(2020) | U | U | Y | Y | N | N | Y | Y | 4 |
| Cai et al.(2020) | Y | Y | Y | Y | N | N | Y | Y | 6 |
| Li et al.(2020) | Y | Y | Y | Y | N | N | Y | Y | 6 |
| Wang et al.(2020) | Y | Y | Y | Y | N | N | Y | Y | 6 |
| Ye et al.(2020) | Y | Y | Y | Y | N | N | Y | Y | 6 |
| Hu et al.（2020） | Y | Y | Y | Y | N | N | Y | Y | 6 |
| Tang et al.（2022） | Y | Y | Y | Y | N | N | Y | Y | 6 |
| Zhang et al.(2021) | Y | Y | Y | Y | N | N | Y | Y | 6 |
| Wang et al.(2020) | Y | U | U | Y | Y | N | Y | Y | 4 |
| Meng et al.(2020) | Y | U | Y | Y | N | N | Y | Y | 5 |
| Liu et al.(2021) | Y | Y | Y | Y | N | N | Y | Y | 6 |
| He et al.(2021) | Y | U | U | Y | Y | N | Y | Y | 4 |
| Ma et al.(2020) | U | U | Y | Y | N | N | Y | Y | 4 |
| Wong et al.(2020) | Y | Y | Y | Y | N | N | Y | Y | 6 |
| Huang et al.(2020) | Y | Y | Y | Y | N | N | Y | Y | 6 |
| Chen et al.(2020) | Y | Y | Y | Y | N | N | Y | Y | 6 |
| Lu et al.(2021) | Y | U | Y | Y | N | N | Y | Y | 5 |
| Jiang et al.(2021) | U | U | Y | Y | N | N | Y | Y | 4 |
| Yang et al.(2020) | Y | U | Y | Y | N | N | Y | Y | 5 |
| Teng et al.(2021) | Y | Y | Y | Y | N | N | Y | Y | 6 |
| Yue et al.(2021) | Y | Y | Y | Y | N | N | Y | Y | 6 |
| Chen et al.(2021) | Y | Y | Y | Y | N | N | Y | Y | 6 |
| Liu et al.(2022) | Y | Y | Y | Y | N | N | Y | Y | 6 |
| Liu et al.(2022) | U | U | Y | Y | N | N | Y | Y | 4 |
| Xu et al.(2021) | Y | Y | Y | Y | N | N | Y | Y | 6 |
| Li et al.(2020) | Y | Y | Y | Y | N | N | Y | Y | 6 |
| Lin et al.(2020) | Y | U | Y | Y | N | N | Y | Y | 4 |
| Xu et al.(2021) | U | U | Y | Y | N | N | Y | Y | 4 |
| Zheng et al.(2021) | U | U | Y | Y | N | N | Y | Y | 4 |

**4.Supplement Table 4:Conversion formulas and references.**

The references and specific formulas for the conversion formula are as follows:

Hogg, R. V., McKean, J., & Craig, A. T. (2005). Introduction to mathematical statistics. Pearson Education.


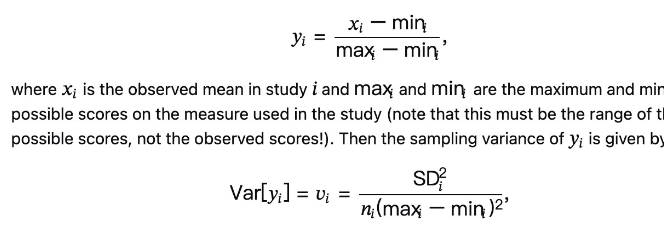

Supplement: Supplementary file 1 [file Data_Sheet_1.docx]
